# Dynamic Causal Models for phase coupling

**DOI:** 10.1016/j.jneumeth.2009.06.029

**Published:** 2009-09-30

**Authors:** W.D. Penny, V. Litvak, L. Fuentemilla, E. Duzel, K. Friston

**Affiliations:** a Wellcome Trust Centre for Neuroimaging, University College, 12 Queen Square, London WC1N 3BG, UK; b Institute for Cognitive Neuroscience, University College, London WC1N 3BG, UK

**Keywords:** Synchronization, Weakly coupled oscillator, Dynamical causal models, MEG, Working memory

## Abstract

This paper presents an extension of the Dynamic Causal Modelling (DCM) framework to the analysis of phase-coupled data. A weakly coupled oscillator approach is used to describe dynamic phase changes in a network of oscillators. The use of Bayesian model comparison allows one to infer the mechanisms underlying synchronization processes in the brain. For example, whether activity is driven by master-slave versus mutual entrainment mechanisms. Results are presented on synthetic data from physiological models and on MEG data from a study of visual working memory.

## 1 Introduction

This paper shows how neuronal synchronization processes, measured with brain imaging data, can be described using weakly coupled oscillator (WCO) models. We expect that these models will be of particular interest to imaging neuroscientists as they can be derived from both bottom-up and top-down principles. The bottom-up approach proceeds by applying a phase reduction approach to neurophysiologically realistic neural network models (Hansel et al., 1995; Ermentrout and Kleinfeld, 2001; Brown et al., 2004). This paper focusses primarily on a top-down approach and uses an inferential framework, Dynamic Causal Modelling (DCM), to fit WCOs to brain imaging data and so make inferences about the structure of neuronal interactions. For example, whether synchronization arises via master-slave or mutual entrainment processes.

At the advent of systems neuroscience Carl Wernicke proposed that complex behaviours are mediated by the interaction of multiple, functionally specialised brain regions. Recent neuroimaging studies suggest that such interactions may be instantiated by the transient synchronization of oscillatory neuronal ensembles (Singer, 1999; Varela et al., 2001; Ward, 2003). For example, contour detection is accompanied by gamma band synchronization in distant parts of visual cortex, multimodal object processing by parieto-temporal synchronization in the beta band (von Stein et al., 1999) and spatial memory processes by hippocampal–prefrontal synchronization in the theta band (Jones and Wilson, 2005).

Questions about entrainment have a long history in studies of circadian and locomotor rhythms (Buzsaki, 2006) and are more recently arising in studies of sleep, memory and higher cognitive processes (Schnitzler and Gross, 2005). A current debate, for example, concerns the origin of the theta rhythm observed during memory-related activity, with some authors proposing a septal-pacemaker hypothesis (Vinogradova et al., 1995) and others that theta arises from septo–hippocampal interactions (Denham and Borisyuk, 2000). More recently, it has been proposed that the interaction of distinct theta processes in medial temporal lobe and hippocampus may provide a substrate for some types of short-term memory (Mormann et al., 2008). The method described in this paper, allied with measurements of multivariate phase time series, will allow one to address such issues empirically. Importantly, one can study phase interactions in a network of regions, thus going beyond simple bivariate analyses.

The weakly coupled oscillator approach we describe is implemented within the Dynamic Causal Modelling (DCM) framework in which a numerical integration scheme, for iterating differential equations, is embedded within a Bayesian estimation and inference framework. Importantly, this allows for different model structures to be compared using Bayesian model selection (Penny et al., 2004). DCMs have so far been developed for event-related potentials (David et al., 2006), steady-state responses (Moran et al., 2009) and changes in induced power (Chen et al., 2008), and this paper extends that domain to phase coupling.

### 1.1 Measuring synchronization

There are currently a host of measures for characterizing synchronization between brain regions (Pereda et al., 2005; David et al., 2004). Perhaps the most well known is the phase locking value (PLV) (Tass et al., 1998; Lachaux et al., 1999). This measures the phase consistency over trials for a certain time-window of interest within which the dynamics are considered to be stationary. Other well known methods involve computation of power spectral density (PSD) matrices via periodogram or autoregressive approaches. One can then compute a number of measures, such as the coherence or phase-lag between regions, or various Granger causality measures expressed in the time or frequency domain. Again, these measures are based on an assumption of signal stationarity during the time-windows of interest.

A contrasting approach is one based on the WCO framework, in which the rate of change of phase of one oscillator is related to the phase differences between itself and other oscillators (Hoppensteadt and Izhikevich, 1997). Thus, because the phases are continually changing the dynamics are considered nonstationary. The parameters of WCO models can be related to biophysically realistic neural network models, using a phase reduction approach (Hansel et al., 1995; Ermentrout and Kleinfeld, 2001; Brown et al., 2004). Additionally, as noted in Rosenblum and Pikovsky (2001), interactions between oscillators can be characterized even if they are too weak to induce full synchronization.

Rosenblum and Pikovsky (2001) have used this framework to derive an empirical measure of coupling direction. This method, referred to in a later paper as the evolution map approach (EMA) (Pikovsky and Rosenblum, 2003), is based on a general linear model (GLM) in which the independent variables are Fourier expansions of the phase differences among regions and the dependent variable is the time derivative of the unwrapped phase in each region. The current paper extends the EMA approach by allowing one to make inferences about network structures involving more than two regions.

The paper is structured as follows. Section 2 briefly reviews the weakly coupled oscillator (WCO) approach and then describes the underlying phase response curves (PRCs) and phase interaction functions (PIFs). We then describe a Dynamic Causal Model for phase coupling based on WCOs. This includes a description of the model itself, the prior distributions, the model fitting and selection procedures, a motivation for our choice of PIF and an analysis of system dynamics. Results are then presented on data generated from bivariate physiological models to assess the robustness of the model estimation and selection approach. We then present results using MEG data acquired during a visual working memory paradigm.

## 2 Methods

### 2.1 Weakly coupled oscillators

The theory of weakly coupled oscillators applies to system dynamics close to limit cycles. By assuming that weak coupling leads to only small perturbations away from these cycles, one can reduce a high-dimensional system of differential equations to one based solely on the phases of the oscillators, and pairwise interactions between them. The theory is based on standard treatments of nonlinear oscillators (Guckenheimer and Holmes, 1983; Nayfeh and Mook, 1979), and the WCO equations are formally derived in Kuramoto (1984), Ermentrout and Kopell (1990), Hoppensteadt and Izhikevich (1997). The aim of this section is to review the main concepts and to sketch a derivation of the equations highlighting what the main assumptions are.

**Fig. 1:**
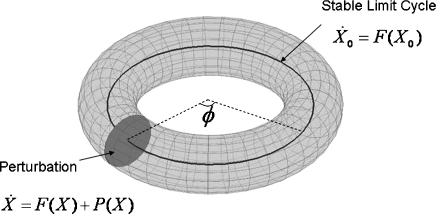
Phase reduction. The solid circular line shows the state space $X_{0}$ of a system on a limit cycle. The limit cycle is assumed stable so that after a small perturbation, the system returns to $X_{0}$. Although $X_{0}$ may be high-dimensional the state will be uniquely determined by its position around the orbit, or the ‘phase’, $\phi \left(X_{0}\right)$. The dynamics of perturbed solutions are constrained to the space *X* shown by the torus. The solid disc corresponds to an ‘isochron’, meaning that all points on this disc have the same asymptotic phase. Using this notion, as we show in the main text, the high-dimensional state equation can be reduced to the one-dimensional system $\dot{\phi }=f+z\left(\phi \right)p\left(\phi \right)$. This is known as a *phase reduction*.

Fig. 1 shows a single oscillator close to a limit cycle. Dynamics on the limit cycle are given by

(1) $$\begin{array}{c} {\dot{X}}_{0}=F\left(X_{0}\right)\\ X_{0}\left(t+T\right)=X_{0}\left(t\right)\\ \dot{\phi }\left(X_{0}\right)=f \end{array}$$

where the dot notation denotes a time derivative and T=1/f is the period of oscillation. Although the dimension of the state space $X_{0}$ may be high, because the system is on a limit cycle its position is uniquely determined by its phase $\phi \left(X_{0}\right)$, i.e. how far it is round the cycle. Dynamics close to the limit cycle are described by

(2) $$\dot{X}=F\left(X\right)+P\left(X\right)$$

where P(X) is a perturbation function. If we define phase, ϕ, in its asymptotic sense (Kuramoto, 1984), i.e. the asymptotic phase difference between two points in *X* is given by their phase difference on the limit cycle after convergence, then by the chain rule of differentiation the rate of change of phase can be expressed as

(3) $$\dot{\phi }\left(X\right)=\frac{d\phi \left(X\right)}{dX}\dot{X}=\frac{d\phi \left(X\right)}{dX}F\left(X\right)+\frac{d\phi \left(X\right)}{dX}P\left(X\right)$$

We now make the assumption that because *X* is close to $X_{0}$ we can evaluate all terms on the right side at $X_{0}$ rather than *X*. This gives

(4) $$\dot{\phi }\left(X\right)\approx \frac{d\phi \left(X_{0}\right)}{dX_{0}}F\left(X_{0}\right)+\frac{d\phi \left(X_{0}\right)}{dX_{0}}P\left(X_{0}\right)$$

The above assumption is a heuristic which requires us to assume that all functions and derivatives are smooth. A more formal derivation based on introducing a smallness parameter into Eq. (2) and taking a geometric perspective, is given in Sections 3.2 and 5.2 in (Kuramoto, 1984). Alternatively, one can derive the WCO equations from a more careful consideration of the required coordinate transforms, as in the appendix of Ermentrout and Kopell (1990). The derivations in Kuramoto (1984) and Ermentrout and Kopell (1990) also require smooth mappings.

Eq. (4) can be rewritten as follows. First, because $X_{0}$ maps on to phase via the function $\phi \left(X_{0}\right)$ we can rewrite $P\left(X_{0}\right)$ with an equivalent function p(ϕ), known as a perturbation function. Second, by the chain rule, the first term is equivalent to the instantaneous frequency on the limit cycle, i.e. f=dϕ/dt. We can therefore rewrite the equation as

(5) $$\dot{\phi }=f+z\left(\phi \right)p\left(\phi \right)$$

where

(6) $$z\left(\phi \right)=\frac{d\phi \left(X_{0}\right)}{dX_{0}}$$

is known as a phase response curve (PRC). We will focus on these two functions in more detail in the following sections.

**Fig. 2:**
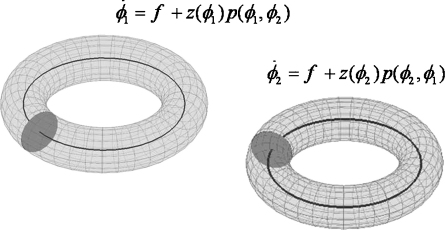
Pair of weakly coupled oscillators. The figure shows two oscillators that are weakly coupled via the perturbation function $p\left(\phi_{1}\text{,}\phi_{2}\right)$. By assuming that the phase difference $\phi =\phi_{1}-\phi_{2}$ changes on a slower time scale than the period of oscillation T=1/f, the right hand side of the above equations can be rewritten as a function solely of phase differences ${\dot{\phi }}_{1}=f+\Gamma \left(\phi_{1}-\phi_{2}\right)$, ${\dot{\phi }}_{2}=f+\Gamma \left(\phi_{2}-\phi_{1}\right)$ where Γ is referred to as the phase interaction function.

The same analysis can be applied to a pair of oscillators (see e.g. page 65 in Kuramoto, 1984), as depicted in Fig. 2, where the perturbation now depends on both phases

(7) $$\begin{array}{c} {\dot{\phi }}_{1}=f+z_{1}\left(\phi_{1}\right)p_{12}\left(\phi_{1}\text{,}\phi_{2}\right)\\ {\dot{\phi }}_{2}=f+z_{2}\left(\phi_{2}\right)p_{21}\left(\phi_{2}\text{,}\phi_{1}\right) \end{array}$$

If we further assume that the phase difference $\phi_{1}-\phi_{2}=\phi$ changes sufficiently slowly, then the ‘phase-offset’ terms can be replaced by a time average over a single cycle. This leads to

(8) $$\begin{array}{c} {\dot{\phi }}_{1}=f+\Gamma_{12}\left(\phi_{1}-\phi_{2}\right)\\ {\dot{\phi }}_{2}=f+\Gamma_{21}\left(\phi_{2}-\phi_{1}\right) \end{array}$$

where

(9) $$\Gamma_{ij}\left(\phi \right)=\frac{1}{2\pi }\int_{0}^{2\pi }z_{i}\left(\psi \right)p_{ij}\left(\psi \text{,}\psi +\phi \right)\;d\psi$$

is known as the ‘phase interaction function (PIF)’. This approach can be extended to a network of $N_{R}$ regions, where the rate of change of phase of the *i*th oscillator is given by

(10) $${\dot{\phi }}_{i}=f_{i}+\overset{N_{R}}{\underset{j=1}{\sum }}\Gamma_{ij}\left(\left(\phi_{i}-\phi_{j}\right)-d_{ij}\right)$$

where $f_{i}$ is the intrinsic frequency of the *i*th oscillator. For $N_{R}=2$ this model is analysed by Earl and Strogatz (2003). We have also introduced an extra phase shift term, $d_{ij}$, (with units of radians). We conceive of this term as arising from a neuronal transmission delay (for a derivation of such phase shifts from neuronal delays see page 259 of Hoppensteadt and Izhikevich, 1997).

To summarize, there are two key assumptions underlying WCOs. The first, that of ‘weak coupling’, assumes that the perturbations are sufficiently small that the functions on the right hand side of Eq. (3) can equivalently be evaluated at $X_{0}$ rather than *X*. The derivation in Kuramoto (1984) makes this explicit via the use of a smallness parameter. The second assumption, that of time-scale separation, is that the relative oscillator phase changes sufficiently slowly, with respect to the oscillation frequency, that the phase-offset term can be replaced by a time average. The WCO framework has been validated, for example, by Hansel et al. (1995) and by Ermentrout and Kleinfeld (2001) using a variety of single neuron models.

### 2.2 A Dynamic Causal Model

We propose a model for narrowband data where signals have been filtered to lie within the band $f_{0}\pm f_{b}$. We consider activity in $N_{R}$ regions and experimental manipulations involving $N_{c}$ ‘modulatory inputs’ or ‘experimental conditions’.

Multiple trials of data per experimental condition will be modelled as this allows for a wider exploration of dynamical state space, which should then lead to better parameter estimation. As an extreme example of using only a single trial, consider one in which the dynamics were already at equilibrium. No phase changes would be observed and it would not be possible to estimate parameters. A further benefit of modelling multiple trials is that, given the larger number of data points, one is able to infer more complex models.

A drawback of modelling multiple trials is the increased amount of computer time required. From this perspective, one would like to model just an ‘average trial’. However, it is difficult to obtain a representative ‘average’ trial that respects pairwise instantaneous phase differences (see Eq. (10)). This issue, that averaging does not preserve information about phase dynamics, is discussed extensively in Tass (2004). We therefore pursue the multi-trial approach. Another possibility here would be to just model trials that had been shown to contain interesting task-related differences, as identified by other methods (Rosenblum and Pikovsky, 2001).

On the *k*th trial, the change of phase in region *i* is given by

(11) $${\dot{\phi }}_{ki}=f_{i}+\overset{N_{R}}{\underset{j=1}{\sum }}\Gamma_{ij}\left(\phi_{ki}-\phi_{kj}\right)$$

where $f_{i}$ is the intrinsic frequency of the *i*th oscillator, $\phi_{ki}$ and $\phi_{kj}$ are the phases in the *i*th and *j*th regions on the *k*th trial. The above equation specifies the rate of change of phase, which is equivalent to the instantaneous frequency. In this paper, delays between regions are not considered explicitly but are rather absorbed into the PIFs (see below and discussion).

In this paper, the PIF is approximated using a Fourier series representation. The motivations for this choice of PIF are discussed in a following section. Additionally, we note that because Γ is a modulo 2π function, expanding it as a Fourier series is topologically equivalent to a Taylor series expansion for non-modulo functions. We have

(12) $$\Gamma_{ij}\left(\phi \right)=-\overset{N_{s}}{\underset{n=1}{\sum }}{ã}_{ijn}^{s}\;\sin\left(n\phi \right)+\overset{N_{c}}{\underset{n=1}{\sum }}{ã}_{ijn}^{c}\;\cos\left(n\phi \right)$$

where changing $N_{s}$ and $N_{c}$ gives us independent control over the number of sine and cosine terms. Our earlier assumption that Γ is smooth now implies that $N_{s}$ and $N_{c}$ are small. The magnitudes of the Fourier coefficients can themselves be changed by ‘modulatory inputs’ as follows

(13) $$\begin{array}{c} {ã}_{ijn}^{s}=|a_{ijn}^{s}+\overset{N_{q}}{\underset{q=1}{\sum }}u_{kq}b_{ijn}^{sq}|\\ {ã}_{ijn}^{c}=|a_{ijn}^{c}+\overset{N_{q}}{\underset{q=1}{\sum }}u_{kq}b_{ijn}^{cq}| \end{array}$$

where $u_{kq}$ is a between-trial experimental variable. For example, for multiple trial types, if trial *k* is of type *q* then $u_{kq}=1$ and is zero otherwise. In the MEG example in Section 2.3 we have k=1,…,20 trials of data which are either ‘control trials’ (q=1) or ‘memory trials’ (q=2). The matrix *U*, with entries $u_{kq}$, then specifies which trials are of which type. The parameters $a_{ijn}^{s}$ and $a_{ijn}^{c}$ are referred to as ‘endogenous’ connectivity parameters and are stored in the matrices $A_{n}^{s}$ and $A_{n}^{c}$, for the *n*th-order odd and even Fourier terms respectively. That is, the *ij*th entry in the matrix $A_{n}^{s}$ is $a_{ijn}^{s}$, and the *ij*th entry in the matrix $A_{n}^{c}$ is $a_{ijn}^{c}$. The parameters $b_{ijn}^{sq}$ and $b_{ijn}^{cq}$ are referred to as ‘modulatory’ parameters and are stored in the matrices $B_{n}^{sq}$ and $B_{n}^{cq}$. This follows the usual DCM nomenclature (Kiebel et al., 2006; Chen et al., 2008). Following Rosenblum and Pikovsky (2001), the magnitude of a connection is given by the norm, ||ã||, which combines odd and even terms.

Following, again, the model in Rosenblum and Pikovsky (2001) the observed time series in region *i* on the *k*th trial is given by the unwrapped phase variable, $y_{ki}$. These time series constitute the data that is to be modelled. The model predictions, $\phi_{ki}$, are generated by integrating Eq. (11) using a Dormand–Prince method (Press et al., 1992). For each trial, the initial phase variables $\phi_{ki}\left(0\right)$ are set equal to the initial observed phases $y_{ki}\left(0\right)$. This provides the starting points for each integration. The statistical model is then given by

(14) $$y_{ki}=\phi_{ki}+e_{ki}$$

where $e_{ki}$ is additive Gaussian noise with zero-mean and covariance matrix $C_{e}\left(\lambda \right)$, and λ are ‘precision parameters’ to be estimated. In this paper, $C_{e}$ is structured so that each region has a different precision parameter, $\lambda_{i}$.

All parameters are concatenated into the vector θ={vec(A),vec(B),f} where vec(X) is a vectorising operator that converts a matrix into a vector by stacking its columns on top of each other (it can be implemented using the Matlab function vec). We also note that it may be useful to include an additive term in the observation model (Eq. (14)), to accommodate any relative-phase offsets, and to then always use sine interaction PIFs. This would be suitable if we wished to model dynamics with a single, but unknown, fixed point (see below).

The absolute function in Eq. (13) constrains the connectivity parameters to be positive. The negative sign in Eq. (12) is then chosen so that, if the PIF is simply a sine function, then the global zero lag (GZL) solution will always be a stable fixed point (see below).

### 2.3 Phase interaction functions and Fourier series

The PIFs are chosen to have the functional form of a Fourier series for a number of reasons. Firstly, for 2π-periodic functions such as Γ(ϕ) the Fourier expansion is topologically equivalent to a Taylor expansion for non-periodic functions. Secondly, this is the choice of basis used in the EMA algorithm (Rosenblum and Pikovsky, 2001). There are a number of further reasons, which are more physiological in nature, and result from consideration of PRCs and PIFs derived from neural network dynamics.

If we assume, for the moment (but see below), that the perturbation term is instantaneous (i.e. that p(ψ,ψ+ϕ) in Eq. (9) is a delta function) then PIFs are identical to PRCs. Further, it is possible to relate PRCs to categories of model defined in dynamical systems theory. One such categorisation is based on bifurcations, that is, parameter changes giving rise to qualitatively different dynamics (Guckenheimer and Holmes, 1983). The codimension of a bifurcation is the number of variables that must be changed to induce it. A supercritical Hopf bifurcation, for example, has codimension one and occurs when a stable equilibrium point changes into a stable oscillation. Importantly, in systems whose dynamics lie close to a supercritical Hopf bifurcation, the PRC has the functional form of a first order Fourier series (Brown et al., 2004). That is, z(ϕ)=acos⁡ϕ+bsin⁡ϕ. Moreover, Hopf bifurcations underly oscillations in many neural network models. For example, from models of theta oscillations in hippocampus (Denham and Borisyuk, 2000) to neural mass models of alpha activity in cortex (Grimbert and Faugeras, 2006). Finally, we note that as most neuronal oscillations are transient, dynamics will most likely lie close to bifurcation points, therefore making the above approximation more robust.

More generally, oscillations may arise from other forms of bifurcation and analytical results deriving PRCs for all codimension one bifurcations that lead to stable oscillations are given in Brown et al. (2004). A further common form, for example, is the saddle node on a limit cycle (SNIC) bifurcation which gives rise to PRCs of the form z(ϕ)=1−cos⁡ϕ.

In general, however, perturbations will not be instantaneous (i.e. p(ψ,ψ+ϕ) will not be a delta function) so the PIF will not be equivalent to the PRC. This is because perturbations are mediated by synaptic effects with finite rise times. The resulting PIF is therefore smooth, thus satisfying the assumptions of Section 2.1. As shown in Van Vreeswijk et al. (1994), for example, even if the PRC has a SNIC form, perturbations via alpha-function synapses will lead to PIFs with a first order Fourier series form.

A further reason for using Fourier PIFs is that this formulation can absorb conduction delays. Given that −asin⁡(ϕ−d)=−acos⁡dsin⁡ϕ+asin⁡dcos⁡ϕ, a pure sine PIF will become a PIF with an additional cos⁡ term. This also motivates the sign of the terms in Eq. (12). Given that we use zero-mean priors (see below), PIFs with longer equivalent delays will be more heavily penalized.

Finally, with sufficient terms, the Fourier series can approximate any arbitrary function. The flexibility of incorporating second-order terms will, for example, be required in the motor physiology example in Section 3.1

### 2.4 Fixed points and stability

One reason that the WCO approach is useful is that analysis of the system dynamics described by Eq. (10) can reveal the synchronization properties of a network of oscillators. This can be studied with the usual analysis tools (Wilson, 1999) and involves finding the fixed points of Eq. (10) and linearizing dynamics around them. Studying synchronization properties is greatly facilitated by phase reduction because a limit cycle in the original model space (Eq. (2)) is equivalent to a fixed point in the corresponding WCO model.

The fixed points (FPs), or equilibrium points, are the values of ϕ for which $\dot{\phi }=0$ and can be identified using any numerical root-finding or minimization algorithm, such as Newton’s method. In the context of phase coupling, the family of points for which the relative phases are constant, are of special interest. These relative phases are given by

(15) ρ=Cϕ

where *C* is an appropriately chosen difference matrix. For example, if ϕ is three-dimensional, and the ‘reference region’ is region 1, then *C* is chosen such that

(16) $$\begin{array}{c} \rho_{1}=\phi_{2}-\phi_{1}\\ \rho_{2}=\phi_{3}-\phi_{1} \end{array}$$

The FPs of interest are then that of the relative-phase system $\dot{\rho }=0$. Of particular interest are the global zero lag (GZL) family of points, ρ=0. This is a state in which all regions are phase locked with all others, at zero lag. Partial zero lag (PZL) states are also of interest. For example, three-region networks with bidirectional connections from a central node (see e.g. the second row in Fig. 8) have been shown to exhibit zero lag synchrony between the outermost regions, even in the presence of long conduction delays (Chawla et al., 2001; Vicente et al., 2008).

The stability of the FPs are then governed by the Jacobian matrix, *J*, where the *ij*th entry is given by $d{\dot{\phi }}_{i}/d\phi_{j}$ evaluated at the FP. If this Jacobian has all negative eigenvalues then the FPs will be stable. For the general case, where the PIF is defined using an *n*th-order Fourier series, as in Eq. (12), off-diagonal elements of the Jacobian are given by

(17) $$J_{ij}=\underset{n}{\sum }n{ã}_{ijn}^{s}\cos\left(n\left(\phi_{i}-\phi_{j}\right)\right)+\underset{n}{\sum }n{ã}_{ijn}^{c}\sin\left(n\left(\phi_{i}-\phi_{j}\right)\right)$$

and diagonal elements by

(18) $$J_{ii}=-\underset{n}{\sum }n{ã}_{ijn}^{s}$$

For the general case of an arbitrary order Fourier series approximation to the PIF, system dynamics can be understood by finding the FPs and then testing for stability, as described above. For three-dimensional systems it is possible to draw state space diagrams in the two-dimensional space ρ. We will provide an example of this in Section 3.2, using a DCM estimated from MEG data. Additionally, there are a number of special cases for which FPs and stability analyses are readily computed. These include bivariate models and sine interactions, discussed below. We also note that in networks without loops, and with first order Fourier PIFs, the FPs can be derived analytically.

#### 2.4.1 Bivariate models

As a simple example of FP and stability analysis, consider a pair of oscillators with $f_{1}=f_{2}$ and identical interaction functions. For any bivariate system the relative-phase space is one-dimensional. That is, we can rewrite Eq. (8) as a single equation involving the phase difference $\rho =\phi_{1}-\phi_{2}$.

(19) $$\dot{\rho }=G\left(\rho \right)$$

where the right hand side of this equation is the odd part of the PIF (assuming $\Gamma_{12}=\Gamma_{21}$)

(20) G(ρ)=Γ(ρ)−Γ(−ρ)

This has stable points at ρ if dG(ρ)/dρ<0. If the PIF is a Fourier series, then stability is determined solely by the odd terms (sine terms). As a simple example if $\Gamma_{ij}=-a\sin\left(\phi_{i}-\phi_{j}\right)+b\cos\left(\phi_{i}-\phi_{j}\right)$, in a bilaterally connected system then G(ρ)=−2asin⁡(ρ). This indicates that synchronization is twice as fast as in the equivalent unidirectional system with a stable fixed point at ρ=0. Ermentrout and Kleinfeld present a similar bivariate stability analysis using PIFs derived from Hodgkin–Huxley models of motor neurons (Ermentrout and Kleinfeld, 2001). In this paper, in Section 3.1 we present an example from motor physiology which specifies first and second-order Fourier forms for G(ρ).

#### 2.4.2 Sine interactions

If the PIF is given simply by the sine operator, that is in Eq. (12) we have $N_{s}=1\text{,}N_{c}=0$, such that $\Gamma_{ij}\left(\phi \right)=-{ã}_{ij1}^{s}\sin\left(\phi \right)$, then the GZL solution is always a stable fixed point. This is the reason why phase oscillators with sine interactions are widely studied (Kuramoto, 1984). In this case, the connectivity parameters, ${ã}^{s}$, can be interpreted such that a stronger connection from *j* to *i* results in $\phi_{i}$ changing more quickly to align with $\phi_{j}$.

**Fig. 3:**
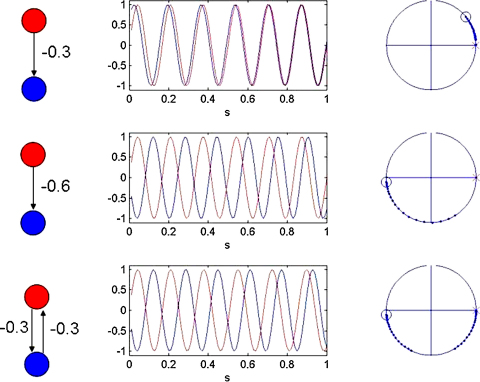
Bivariate sine interactions. The left column shows the network structure used to generate the data in each row. The middle column shows the corresponding bivariate time series for two oscillators, $\sin\left(\phi_{1}\right)$ (red) and $\sin\left(\phi_{2}\right)$ (blue). The right column shows the corresponding phase diagrams on the unit circle with initial phases marked as a red cross for the first oscillator, and as a blue circle for the second. Subsequent phase evolutions are shown using dots. These data were generated from bivariate WCO models with sine interaction functions ${\dot{\phi }}_{1}=f+a_{12}\sin\left(\phi_{1}-\phi_{2}\right)$ and ${\dot{\phi }}_{2}=f+a_{21}\sin\left(\phi_{2}-\phi_{1}\right)$. Different rows correspond to data generated using different model parameters and/or initial phases. The first row was produced using $a_{12}=0$, $a_{21}=-0.3$, the second row $a_{12}=0$, $a_{21}=-0.6$ and the third $a_{12}=-0.3$, $a_{21}=-0.3$. In all cases negative *a* values move the system towards zero lag synchronization, the absolute value of *a* indicating the speed of convergence. For the first row oscillator 2 slows down. In the second row, due to the different initial conditions oscillator 2 speeds up. In the third row oscillator 2 speeds up and oscillator 1 slows down. (For interpretation of the references to color in this figure legend, the reader is referred to the web version of the article.)

This is demonstrated in Fig. 3 for three different bivariate WCO models. In all cases negative *a* values move the system towards zero lag synchronization, the absolute value of *a* indicating the speed of convergence. For the first model (first row in Fig. 3) synchronization is achieved by oscillator 2 slowing down. In the second model, due to the different initial conditions, synchronization is achieved by oscillator 2 speeding up. In the third model, oscillator 2 speeds up and oscillator 1 slows down. In all of these models the anti-phase state is an unstable fixed point.

### 2.5 Priors

We use the following priors on the model’s unknown parameters θ={A,B,f}

(21) $$\begin{array}{c} p\left(a_{ijn}^{s}\right)=N\left(0\text{,}\sigma_{p}\right)\\ p\left(a_{ijn}^{c}\right)=N\left(0\text{,}\sigma_{p}\right)\\ p\left(b_{ijn}^{s}\right)=N\left(0\text{,}\sigma_{p}\right)\\ p\left(b_{ijn}^{c}\right)=N\left(0\text{,}\sigma_{p}\right)\\ p\left(f_{i}\right)=N\left(f_{0}\text{,}\sigma_{f}\right) \end{array}$$

where *A* and *B* are the endogenous and modulatory parameters describing the phase interaction functions, and *f* is a vector of intrinsic oscillation frequencies. As the data are bandlimited, we know that the instantaneous frequency lies between $f_{0}\pm f_{b}$. We set $\sigma_{p}=f_{b}/3.3$ so that the probability that the instantaneous frequency (see Eq. (11)) lies outside this range, due to any single contribution of the above parameters, is less than p=0.001. We consider two different priors on the intrinsic frequencies. A ‘hard prior’ is given by $\sigma_{f}=10^{-6}$ which effectively constrains all frequencies to $f_{0}$. A ‘soft prior’ is given by $\sigma_{f}=0.1\sigma_{p}$ and allows for small variations. In our software implementation (see later) the right hand side of Eq. (11) is multiplied by 2π so that the units of frequency are Hertz, rather than radians per second.

### 2.6 Model estimation

For a given DCM, say model *m*, parameter estimation corresponds to approximating the posterior distribution given by Bayes rule

(22) $$p\left(\theta |y\text{,}m\right)=\frac{p\left(y|\theta \text{,}m\right)p\left(\theta |m\right)}{p\left(y|m\right)}$$

The estimation procedure used is described in detail in Friston (2002). The posterior moments are updated iteratively using Variational Bayes under a Gaussian approximation to the posterior density q(θ|m)=N(μ,Σ). This is equivalent to Expectation-Maximization (EM) that employs a local linearization of the predicted responses about the current posterior expectation of the parameters. In the ‘E-step’ the variational free energy F(q,λ,m) is optimized with respect to the moments of *q* and in the ‘M-step’, it is optimized with respect to the precision parameters λ. The estimation scheme can be summarized as follows

(23) $$q_{\text{new}}=\underset{q}{arg max}F\left(q\text{,}\lambda \text{,}m\right)$$

(24) $$\lambda_{\text{new}}=\underset{\lambda }{arg max}F\left(q\text{,}\lambda \text{,}m\right)$$

where the *q* update is known as the E-step and the λ update as the M-step. These two steps are iterated until convergence. The free energy is given by

(25) F(q,λ,m)=log⁡p(y|λ,m)−KL(q(θ)||p(θ|y,λ,m))

This equation shows that the free energy is equivalent to the log-evidence minus the Kullback–Leibler divergence between the real and approximate posterior density. This means that the optimization maximizes the log-evidence, while minimizing the discrepancy between the true and approximate posteriors. This scheme is identical to that employed by other DCMs (David et al., 2006; Chen et al., 2008).

### 2.7 Model comparison

Inference on the parameters of a particular model uses the approximate posterior density, q(θ|m). Usually, this involves specifying a parameter or compound of parameters as a contrast, $c^{T}\mu$. Inferences about this contrast are made using its posterior covariance, $c^{T}\Sigma c$. For example, in a model having three parameters, one can test if the first is larger than the second using the contrast weights $c^{T}=\left[1\text{,}-1\text{,}0\right]$. This inference is conditioned on the particular model specified. However, in many situations one wants to compare different models, for example master-slave versus mutual entrainment models. This entails Bayesian model comparison in which different models are compared using their evidence (Penny et al., 2004). The model evidence is

(26) p(y|m)=∫p(y|θ,m)p(θ|m)dθ

and can be decomposed into an accuracy term, which quantifies the data fit, and a complexity term, which penalizes models with a large number of parameters. In the following, we approximate the evidence for model *m*, with its free energy bound

(27) log⁡p(y|m)≈F(m)

The most likely model is the one with the largest log-evidence. Model comparison rests on the likelihood ratio of the evidence for two models which is known as the Bayes factor $B_{ij}$ where

(28) $$\begin{array}{c} \log B_{ij}=\ln\frac{p\left(y|m=i\right)}{p\left(y|m=j\right)}=F\left(m=i\right)-F\left(m=j\right) \end{array}$$

Conventionally, strong evidence in favour of one model requires the difference in log-evidence to be about three or more (i.e. a Bayes factor of about 20). In what follows, we will compare models with different structures. By assuming uniform priors on the models we can convert the model evidence into a posterior probability over models by normalizing the evidences so that they sum to one. Under this assumption, two models with a log-evidence difference of three imply that we can be 95% confident that the better model is more likely, given the data. This follows from Bayes rule

(29) $$p\left(m|y=i\right)=\frac{p\left(y|m=i\right)p\left(m=i\right)}{p\left(y|m=i\right)p\left(m=i\right)+p\left(y|m=j\right)p\left(m=j\right)}$$

and by noting the priors are uniform and substituting p(y|m=j)=p(y|m=i)exp(−3).

The model comparisons of primary concern in this paper are inferences about connections between regions. For example, whether the coupling that brings about synchronization is unidirectional or bidirectional. In this paper, we also refer to unidirectional coupling as ‘master-slave’ coupling, and to bidirectional coupling as ‘mutual entrainment’. This is simply because with unidirectional coupling, the sink (receiving) region changes its phase to align with the source region. The source therefore acts as a master and the sink as a slave. With bidirectional coupling both regions change their phase to align with each other. We note that in synergetics (Haken et al., 1985), the term master-slave is used somewhat differently, and refers to a separation of time scales. An example of applying DCM for phase coupling in a synergetics context is given in Section 3.1.

### 2.8 Application to M/EEG

For the application of the algorithm to M/EEG, the data are preprocessed as follows. First, the data are bandpass filtered into the range of interest. Given that we are interested in reconstructing activity in $N_{R}$ source regions with known anatomical locations, we can form the $N_{S}\times N_{R}$ lead field matrix *L* where $N_{S}$ is the number of M/EEG sensors. The source locations can be identified from previous studies, or by source reconstruction of event-related potentials (ERPs) (Friston et al., 2008) or induced power activity (Friston et al., 2005; Sekihara et al., 2002).

Given that $N_{R}<N_{S}$, activity in the $N_{R}$ regions can be estimated via the maximum likelihood projection (Baillet et al., 2001)

(30) $$y\left(k\right)=L^{-}y_{sens}\left(k\right)$$

where $L^{-}$ denotes a generalized inverse, $y_{sens}\left(k\right)$ is the $N_{s}\times T$ data matrix in sensor space and *k* indexes the trial number. This is the same projection as is used in DCM for induced responses (Chen et al., 2008). This data is then subjected to a Hilbert transform to extract the instantaneous phase in each region. The phases are then unwrapped by changing absolute jumps greater than or equal to π to their 2π complement.

## 3 Results

### 3.1 Synthetic bimanual finger movements

This section applies DCM for phase coupling to data generated from an abstract physiological model of bimanual finger movement proposed by Haken et al. (1985). This model was motivated by the following phenomenon. If you move your left and right index fingers, initially slowly and in *anti-phase* with each other, then as you gradually increase the frequency your fingers will eventually move *in-phase*. This change of behaviour, from anti-phase to in-phase, can be characterized by a *G* function (the odd part of the PIF) of the form

(31) G(ϕ)=−asin⁡ϕ−b(f)sin⁡2ϕ

where $\phi =\phi_{1}-\phi_{2}$ is the phase difference between fingers, and the second coefficient b(f) is a decreasing function of frequency *f*. For small *f* there are two stable equilibrium points, in-phase ϕ=0 and anti-phase ϕ=π. As *f* is increased beyond the critical value b(f)=a/4 the anti-phase attractor becomes unstable.

In the language of ‘synergetics’ (Haken et al., 1985)b(f) is an ‘order’ parameter and it is helpful to think of G(ϕ) as the derivative of a potential function V(ϕ). That is

(32) $$G\left(\phi \right)=-\frac{dV}{d\phi }$$

(33) V(ϕ)=−acos⁡ϕ−b(f)cos⁡2ϕ

**Fig. 4:**
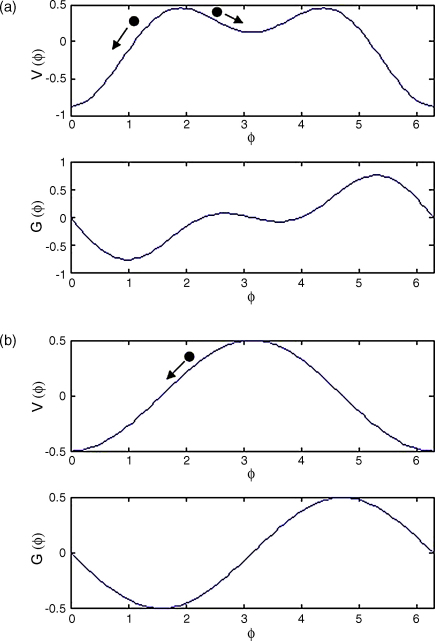
Bimanual finger movement. Potential functions V(ϕ) and phase interaction functions G(ϕ) for (a) low frequency and (b) high frequency bimanual finger movement. The phase difference $\phi =\phi_{1}-\phi_{2}$ changes by following the gradient of the potential function $\dot{\phi }=-dV/d\phi$ (see filled circles and arrows). At low frequency, both in-phase (ϕ=0) and anti-phase (ϕ=π) minima are stable, whereas at high frequency only the in-phase minimum is stable.

The WCO equations can then be thought of as following the gradient of the potential function to a local minimum. As shown in Fig. 4(a) there are two such minima at low frequency. At high frequency, shown in Fig. 4(b), the anti-phase minimum disappears resulting in the observed phenomenon.

We use this simple model as a test bed for the DCM method. We generate bivariate time series for the phase of left, $\phi_{L}$, and right index fingers, $\phi_{R}$, using the following equation:

(34) $$\begin{array}{c} {\dot{\phi }}_{L}=f\\ {\dot{\phi }}_{R}=f+\Gamma \left(\phi_{R}-\phi_{L}\right) \end{array}$$

where for the unimodal (U) model

(35) Γ(ϕ)=−0.5sin⁡ϕ

and for a high frequency or bimodal (B) model

(36) Γ(ϕ)=−0.5sin⁡ϕ−0.375sin⁡2ϕ

These equations model a left-finger oscillator with fixed phase (relative to oscillation at frequency *f*), and a right finger oscillator that changes phase as a function of the phase difference, $\phi_{R}-\phi_{L}$.

**Fig. 5:**
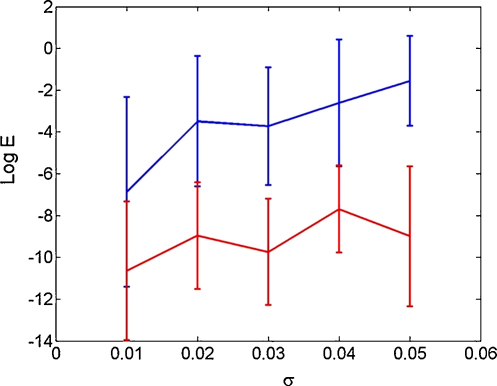
DCM versus EMA. The figure plots the log of the parameter estimate error (mean and 1SD error bars), versus the observation noise level, σ, for the DCM (red) and EMA (blue) estimation methods. (For interpretation of the references to color in this figure legend, the reader is referred to the web version of the article.)

We first compare the accuracy of DCM and the evolution map approach (EMA) (see Section 1) estimation methods by generating a single trial of data from the U model, and adding observation noise of standard deviation σ to the unwrapped phases. We chose, arbitrarily, f=6 Hz and created 1s data with a sample rate of 100 Hz (for our purposes, the absolute value of the frequency is irrelevant). We estimated U model parameters using DCM and EMA and, for each, computed the parameter estimation error, $E={\left(â-a\right)}^{2}$, where the true parameter value a=0.5 and â is the estimated value. In the DCM implementation we used soft priors on the intrinsic frequencies. Fitting was repeated for 20 data sets at each noise level. Fig. 5 plots log⁡E (mean and 1SD error bars) versus σ for each method, showing DCM to be significantly more accurate.

**Fig. 6:**
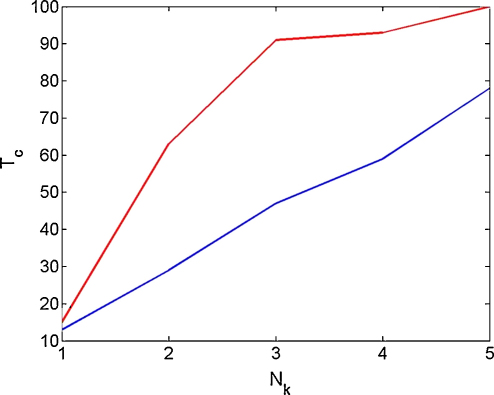
Multiple trials. The figure plots the total number of correct model comparisons, $T_{c}$, out of 100, versus the number of trials used in making each comparison, $N_{k}$, for two different sets of initial conditions. For the red curve the initial phase difference was drawn from a uniform distribution between 0 and 2π, and for the blue curve from a uniform distribution between −2 and 2. (For interpretation of the references to color in this figure legend, the reader is referred to the web version of the article.)

The next simulation considers model identification using DCM based on multiple trials of phase data. We generated $N_{K}$ trials of bivariate phase time series from the B model. For each trial, the initial phase was chosen at random from a uniform distribution between 0 and 2π. For each data set we then fitted both a B and a U model, and computed the model evidences F(m=B) and F(m=U). This was repeated 100 times for each $N_{K}$. Fig. 6 (red curve) plots the total number of correct model comparisons, $T_{c}$ versus the number of trials used in making each comparison, $N_{k}$. A model comparison was deemed correct if F(m=B)−F(m=U)>3. This shows that, as is to be expected, the correct model can be identified more frequently if we have more trials of data.

We then repeated this simulation but with the initial phase distribution chosen as uniform within the more restricted range −2≤ϕ≤2. With this initial phase distribution, system dynamics will nearly always converge to the in-phase stable state and it should be more difficult to correctly identify the B model (which additionally allows for stable anti-phase locking). This is indeed what the results in Fig. 6 (blue curve) show. This indicates that the use of multiple trials allows for a wider exploration of state space, and so to a more accurate model identification.

### 3.2 MEG data

**Fig. 7:**
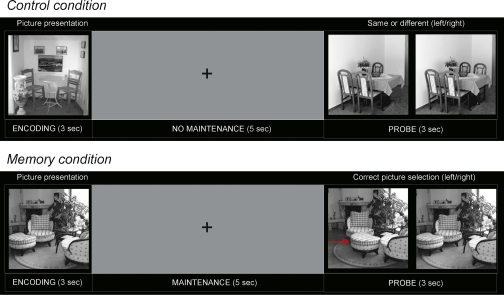
Experimental paradigm. MEG data was acquired during a working memory task using pictures of visual scenes. After a 1 s inter-trial-interval, a visual scene was presented for 3 s (encoding). This was followed by a blank screen with a fixation cross for 5 s (delay) and then by two test stimuli for 3 s (probe). For ‘memory’ trials subjects were required to press a button at probe indicating which of the two test pictures was presented during encoding. For ‘control’ trials, the button-press at probe indicated whether the probe images were the same or different.

This section describes an application to MEG data recorded during a working memory experiment (Fuentemilla et al., submitted for publication). The experimental paradigm, depicted in Fig. 7, consisted of a visual Delayed-Matched-to-Sample task with the use of indoor and outdoor scene pictures. After a 1 s inter-trial-interval, an indoor or an outdoor scene was presented for 3 s (encoding period). This was followed by a blank screen with a fixation cross for 5 s (delay period) and then by two test stimuli for 3 s (probe period). For ‘memory’ trials^1^ subjects were required to press a button at probe indicating which of the two test pictures was presented during encoding. For ‘control’ trials, the button-press at probe indicated whether or not the probe images were different. Control trials did not therefore require a memory trace to be held during the delay period (i.e. no ‘maintenance’ of memory). MEG data were recorded using a 275-channel CTF Omega whole cortex magneto-meter (VSM MedTech, Coquitlam, BC, Canada) with a 480 Hz sampling rate and 300 Hz low pass filtering. Data were then filtered between 4 and 8 Hz (the ‘theta band’) using a zero-phase bandpass filter.

Activity was reconstructed using a maximum likelihood projection (as described in Section 2.8) for three sources in the right hemisphere, denoted (i) MTL with Talairach coordinates (27,−18,−27) mm, (ii) Occ (10,−100,0) mm and (iii) IFG (39,28,−12) mm. Unwrapped phase time series were computed as described in the earlier section on ‘Application to M/EEG’. These three regions were chosen as they showed prominent activity in a source reconstruction, using the Multiple Sparse Priors algorithm (Friston et al., 2008), of event-related components during the probe period (specifically at t=400 ms post-probe).

**Fig. 8:**
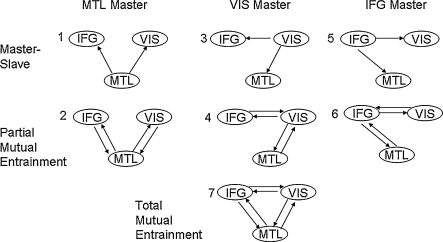
Hypothesized model structures. Theta activity observed using MEG during the delay period of a working memory task is hypothesized to arise from master-slave, partial mutual entrainment or total-mutual entrainment mechanisms.

We extracted 10 trials of control data and 10 trials of memory data, from the first second of the maintenance period. The initial phase vector for each trial was set equal to the observed phase on that trial. We then fitted a number of DCMs, with architectures shown in Fig. 8, to see if phase coupling was mediated by (i) master-slave (models 1, 3 and 5), (ii) partial mutual entrainment (models 2, 4 and 6) or (iii) total-mutual entrainment (model 7) mechanisms. We used first-order Fourier series ($N_{s}=1\text{,}N_{c}=1$) for the PIFs and hard priors (see Section 2.5) for the intrinsic frequencies. We allowed modulatory inputs (i.e. the memory task) to change both sine and cos terms in the PIF (see Eq. (13)). This allows both the fixed points to change, and the speed at which they are reached.

**Fig. 9:**
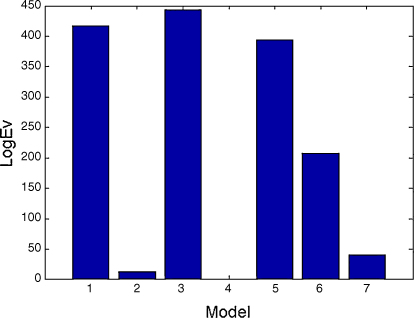
Model comparison. This bar graph plots the log model evidence (relative to the worst model, model 4) for each model structure in Fig. 8. It shows that model 3, in which occipital cortex enslaves activity in IFG and MTL is the most likely cause of synchronized theta activity during maintenance.

As shown in Fig. 9, model 3 had the highest model evidence. The log-evidence difference between model 3 and the next best model, model 1, is F(m=3,F1)−F(m=1,F1)=27.0. This suggests that phase coupling is mediated by occipital cortex driving enslaved oscillations in MTL and frontal cortex. We additionally fitted a sine interaction model and a second-order Fourier series model both with the same structure as model 3, but the first-order Fourier series model had higher evidence (F(m=3,F1)−F(m=3,Sine)=78.8,F(m=3,F1)−F(m=3,F2)=6.7).

**Fig. 10:**
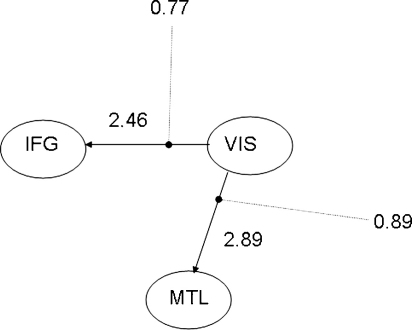
Network parameters. The numbers next to the arrows indicate estimated values of the intrinsic connections (ã in Eq. (13)). The lines ending in filled circles indicate modulatory connections, and the numbers at the end of them show the estimated values ($\tilde{b}$ in Eq. (13)). This follows the usual DCM network diagram semantics. A larger connection value denotes that the receiving region changes its phase more quickly.

Fig. 10 shows the estimated parameter magnitudes for model 3. These magnitudes are computed using the norm of the estimated sine and cos terms (see earlier). A larger connection value denotes that the receiving region changes its phase more quickly. The figure shows that both connections are modulated by the memory task.

**Fig. 11:**
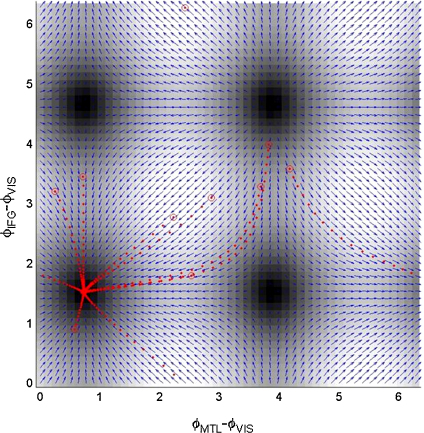
Control. This figure presents a state-space diagram of the estimated phase dynamics for the ‘control’ MEG data. The blue arrows show the flow vector $\dot{\rho }=C\dot{\phi }$ and the background grey scale maps the magnitude $\left||\dot{\rho }|\right|$. The red dots show the fitted trajectories of the ten control trials, with initial values marked with open red circles. (For interpretation of the references to color in this figure legend, the reader is referred to the web version of the article.)

**Fig. 12:**
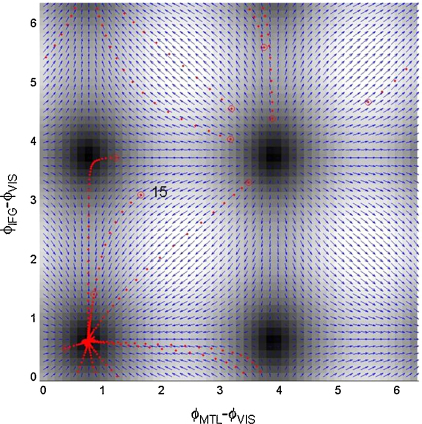
Memory. This figure presents a state-space diagram of the estimated phase dynamics for the ‘memory’ MEG data. The blue arrows show the flow vector $\dot{\rho }=C\dot{\phi }$ and the background grey scale maps the magnitude $\left||\dot{\rho }|\right|$. The red dots show the fitted trajectories of the 10 memory trials, with initial values marked with open red circles. One can see that the FPs have moved, as compared to Fig. 11. The number 15 marks the start of the trajectory of the k=15 th trial (the 5th memory trial), which is also shown in time series format in Fig. 13. (For interpretation of the references to color in this figure legend, the reader is referred to the web version of the article.)

The system dynamics underlying model 3 are perhaps best understood by the state-space diagrams shown in Fig. 11, for the control trials, and Fig. 12, for the memory trials. There are four fixed points in each diagram. A stability analysis based on the system Jacobian at these points (or a visual inspection looking for converging arrows), shows that only the lower-left point is stable. Moreover, this stable fixed-point moves such that the phase difference between IFG and VIS is smaller for the memory than the control trials. Additionally, from the magnitudes in Fig. 10 we know that the fixed point is reached more quickly.

**Fig. 13:**
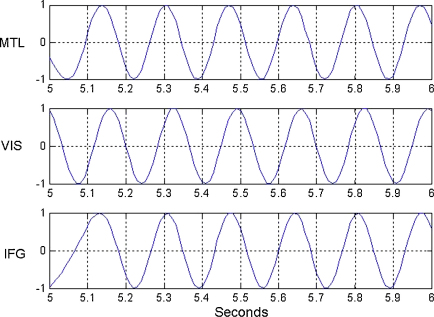
Exemplar trial. This figure shows the fitted time series for the k=15 th trial (the 5th memory trial), plotted as $\sin\phi_{i}$, for data in the MTL, VIS and IFG regions during the first second of the delay period. This trial is also shown in state-space format in Fig. 12. One can see how activity in the different regions becomes synchronized.

Fig. 13 shows the fitted time series for the 15th trial of data (the 5th memory trial). One can see how activity in the different regions gradually becomes synchronized. Of particular interest is the almost zero-lag synchronization at t=6 between regions MTL and IFG. One can also infer this from the stable fixed point for the memory dynamics shown in Fig. 12 where $\phi_{\text{IFG}}-\phi_{\text{VIS}}=0.61$, $\phi_{\text{MTL}}-\phi_{\text{VIS}}=0.76$. This means that $\phi_{\text{MTL}}-\phi_{\text{IFG}}=0.15$ which corresponds to a time offset of 4 ms. The corresponding time offset for the control dynamics is 21 ms.

## 4 Discussion

This paper has presented an extension of the DCM framework to the analysis of phase-coupled data in which a weakly coupled oscillator approach is used to describe dynamic phase changes in a network of oscillators. The WCO approach accommodates signal nonstationarity by using differential equations which describe how changes in phase are driven by pairwise differences in instantaneous phase. This is to be compared with data analysis approaches, such as the PLV or measures derived from autoregressive modelling, which assume signal stationarity.

Previous work on fitting WCO models to experimental data has relied on an evolution map approach (EMA) which is restricted to bivariate data (Rosenblum and Pikovsky, 2001). A more recent approach considers multivariate data but is restricted to isotropic coupling (Tokuda et al., 2007). We have shown using simulations that, for bivariate data, the DCM approach is more accurate than EMA. More importantly, DCM can be applied to networks with more than two regions. Inferences can then be made about network structures giving rise to the observed dynamics. For example, whether synchronization processes depend on master-slave (unidirectional) or mutual entrainment (bidirectional) mechanisms.

We have proposed a DCM approach in which each experimental condition of interest is represented using multiple trials of data. This is to be contrasted with DCM for ERPs (David et al., 2006), for example, in which multiple trials of data are first averaged and then presented to DCM. In the context of phase coupling, the use of multiple trials is necessary so that a range of initial conditions, and therefore different domains of state space, are explored. We have shown using simulations that this leads to improved model inference. Such an approach is not of use in DCM for ERPs, as the underlying dynamical model operates around a known fixed point (zero activation), which is equal to the initial condition.

In the application of DCM to the MEG data, a first order series was used to approximate the PIFs, in which both even and odd terms were allowed to vary between conditions. This allowed DCM to identify both the Fixed Points (FPs; see e.g. Figs. 11 and 12) and dynamics that led to them. In other applications, the FPs may be know a priori, in which case these values can be subtracted from observed data, and DCMs employed using simpler sine interaction PIFs. It may be that these FPs can be identified using multivariate phase clustering methods (Hutt et al., 2003). A third option here would be to include an additive term in the observation model (Eq. (14)), to accommodate any relative-phase offsets, and to then always use sine interaction PIFs. This would be suitable if we wished to model phase dynamics with a single, but unknown, stable fixed point.

Although the use of MEG data in this paper is only aimed at a proof of principle for the methodology, the analysis we have performed does appear consistent with findings from rodent neurophysiology in which fronto–cortical neurons were found to spike at consistent phases of the hippocampal theta rhythm, presumably for the transfer of phase-coded information (Jones and Wilson, 2005). In this paper, in a working memory task, we found that phase dynamics in the memory condition led to near zero-lag correlation between theta activity in fronto–temporal regions. This was not the case for the dynamics in the control condition. Moreover, we can infer that this synchronization was brought about by a master-slave structure in which phase changes in both temporal lobe and frontal cortex were driven by activity in visual cortex.

The following subsections address a number of shortcomings of the current modelling approach, which we hope to address in future publications. We first note that an obvious drawback of the WCO approach is that it ignores amplitude variations, and so provides an incomplete description of brain dynamics. If, however, one is explicitly interested in amplitude changes that give rise to experimentally induced changes of spectral energy then a recent alternative DCM approach may be of use (Chen et al., 2008).

### 4.1 Neurophysiological models

The PIFs which parameterise WCOs can be related to neurophysiologically realistic neural network models in a number of ways. In this paper we have considered categorisations of models from dynamical systems theory, in which models are classified according to the type of bifurcation underlying oscillatory behaviour. This leads to specific forms of PRC and PIF, and motivated us to use Fourier expansions for the PIF.

The connection between WCOs and underlying neurophysiological models can, however, be made more explicit. Indeed, given any differential equation model of neuronal or network activity, in which the system operates around a stable limit cycle, PRCs can be numerically evaluated using perturbation or adjoint methods. These are implemented in the XPP or MATCONT software packages (Govaerts and Sautois, 2006; Ermentrout and Kleinfeld, 2001). Additionally, a simple way of seeing how changes in biological parameters, $\theta_{b}$, affect network synchronization is then via the derivatives $d\Gamma /d\theta_{b}$ where Γ is the PIF (Govaerts and Sautois, 2006). Use of such derivatives will be investigated in future work.

### 4.2 Stochastic dynamics

Another way to extend the biological validity of the WCO approach is to model the evolution of, not just a single phase variable in each region $\phi_{i}$, but a probability density over phases $p\left(\phi_{i}\right)$. These densities can be considered as arising from multiple oscillators within a region and can be specified as solutions of weakly coupled oscillator dynamics based on stochastic differential equations (SDEs). Such an approach has been considered by Brown et al. (2004) who derive analytic results for a population of oscillators with different initial phases, responding to transient inputs. Such stochastic dynamics can be characterized using Fokker–Planck equations (Daffertshofer, 1998) or approximated using moment-closure methods (Ly and Tranchina, 2007; Marreiros et al., 2009). Incorporating such behaviour would require extending the current DCM from deterministic differential equations (DDEs) to SDEs.

### 4.3 Phase resetting

Tass (2003) has also specified weakly coupled oscillator dynamics using SDEs in which experimental inputs give rise to responses which are transiently synchronized over trials. Here the population of responses is over trials, rather than over multiple oscillators within a single trial. Such dynamics can be accommodated by adding an extra term to Eq. (11) describing effects of within-trial inputs $u_{m}$. Following (Tass, 2003) this could take the form $u_{m}c_{im}cos\left(\phi_{i}\right)$ which, provided the input parameters $c_{im}$ were sufficiently large, would cause the phase (over trials) to lock at a certain peri-stimulus time point. This would, as suggested by Makeig et al. (2002), provide a mechanism for the generation of ERF/P components in which system dynamics operate around limit cycles, rather than fixed points as in previous work (David et al., 2006).

### 4.4 Conduction delays

In this paper, conduction delays have been absorbed into the representation of the phase interaction function, using a Fourier series approach. In future we will make use of independent sources of information about conduction delays, such as from diffusion imaging or from anatomical databases as in Ghosh et al. (2008).

## Footnotes

1 These trials correspond to the ‘Configural’ condition as described in Fuentemilla et al. (submitted for publication).
